# Sequential BN-doping induced tuning of electronic properties in zigzag-edged graphene nanoribbons: a computational approach†

**DOI:** 10.1039/c8ra00386f

**Published:** 2018-03-19

**Authors:** Amrit Sarmah, Pavel Hobza

**Affiliations:** Institute of Organic Chemistry and Biochemistry of the Czech Academy of Sciences Flemingovo nam. 2, CZ-16610 Prague 6 Czech Republic amrit.sarmah@marge.uochb.cas.cz +420 731015016; Department of Physical Chemistry, Palacký University CZ–77146 Olomouc Czech Republic

## Abstract

We employed first-principles methods to elaborate doping induced electronic and magnetic perturbations in one-dimensional zigzag graphene nanoribbon (ZGNR) superlattices. Consequently, the incorporation of alternate boron and nitrogen (hole–electron) centers into the hexagonal network instituted substantial modulations to electronic and magnetic properties of ZGNR. Our theoretical analysis manifested some controlled changes to electronic and magnetic properties of the ZGNR by tuning the positions (array) of impurity centers in the carbon network. Subsequent DFT based calculations also suggested that the site-specific alternate electron–hole (B/N) doping could regulate the band-gaps of the superlattices within a broad range of energy. The consequence of variation in the width of ZGNR in the electronic environment of the system was also tested. The systematic analysis of various parameters such as the structural orientations, spin-arrangements, the density of states (DOS), band structures, and local density of states envisioned a basis for the band-gap engineering in ZGNR and attributed to its feasible applications in next generation electronic device fabrication.

## Abstract 2

Incorporation of an alternate impurity array in the ZGNR don't break the spin-degeneracy, providing the freedom to tune the electronic behaviors without affecting the spin-dependent properties.

## Graphical abstract

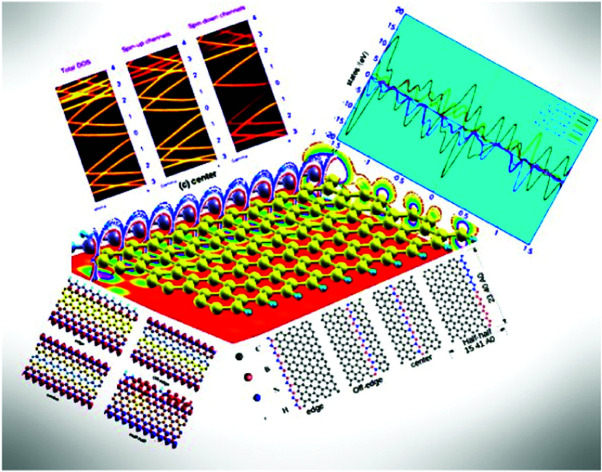

## 1. Introduction

Low dimensional materials adduce tunable electronic and magnetic properties, ensuing electron confinement effects that are highly sensitive to their structural topology and geometrical orientations.^1,2^ Graphene nanoribbons (GNRs) are one-dimensional structures. These thin, elongated strips of graphene showcase extraordinary electronic properties suitable for nanoelectronics and device fabrications. In principle, a graphene nanoribbon (GNR) is a finite-size structure, which is a part of an infinite hexagonal two-dimensional carbon network consisting of small band gaps due to the confinement of electrons in the transverse direction.^3^ Indeed, due to the existence of prolific edges-states, graphene nanoribbons (GNRs) can alter the intrinsic conductivity states from semiconductors to semimetals according to the respective changes in the system's width. Consequently, GNRs are considered to be an extremely versatile variant of graphitic carbon.^4^ The structures of GNRs along with their electronic and magnetic properties have been extensively explored both experimentally and theoretically. Contemporary studies have accounted some good correlations between the structural topology of GNR edges (armchair or zigzag) and their electronic behaviors.^5–7^ Interestingly, the localized edge states formed in zigzag-edged graphene nanoribbons (ZGNRs) show ferromagnetically coupled interactions inside one edge, while showing antiferromagnetic couplings between the two edges. In order to customize the functionality of GNRs, various techniques are adopted, namely, doping, edge modifications in terms of chemical and physical means, and application of an electrical field.^5,8–13^ From experimental prospects, obtaining an adequately stable structure of the system is the fundamental objective. Wassmann *et al.* extensively investigated the energetics and structures of different modeled hydrogen-terminated graphene-nanoribbon edges as a function of hydrogen content of the environment.^14^ The same basic organic chemistry concept also predicted the strong influence of the inherent benzenoid character of the ribbons on the electronic blueprint of GNRs.^15^ To be more precise, Clar's theory of the aromatic sextet demonstrated the potential to provide reasonable predictions on the stability, geometrical parameters, electronic environment, and magnetic properties of graphene nanoribbons containing different hydrogen edge terminations.^16–19^

In the recent years, there was an exponential growth in the research based on edge decorated ZGNR (in both experimental and theoretical aspects). Mostly, these quality studies were immaculately dedicated for understanding the electronic and magnetic properties of ZGNR and the possibilities to tailor these properties for nano-optics and spintronics. In fact, the magnetic ground state associated with ZGNRs display antiferromagnetic coupling between the spin-polarized edge states and show degenerate spin-resolved band structures, resulting in a zero-magnetic moment associated with some non-spin-polarized transport.^20,21^ It is worth mentioning that the implicit spin degeneracy of ZGNR system has to be broken in order to acquire the spin-polarized transport through ZGNRs. As the magnetism or spin-polarized features of ZGNRs directly pertain to the edge states, the best possible solution would be the edge states modification *via* different approaches.^22^ It is apparent from the preceding discussions that the magnetization of ZGNRs can be tuned by means of some external perturbations such as magnetic fields or chemical modifications. The impurity doping at the zigzag edges is educed to be more stable and consequently induce immanent changes to the spin polarity.^23,24^ In a recent study, Huang *et al.* assayed the thermal spin-transport properties of hybrid boron, nitrogen co-doped ZGNR under various magnetic configurations.^25^ In a combined experimental and theoretical study, Makarova *et al.* ascertained the strongly coupled magnetic states at the graphene–fluorographene interface from magnetic susceptibility measurements.^26^ Subsequently, Chen *et al.* impressively demonstrated the effect of metal substrates on the local magnetic moments of zigzag graphene nanoribbons (ZGNRs).^27^ In another important study, Zheng *et al.*^28^ examined concatenated changes in the characteristic ZGNRs band gaps from metallic, semiconducting, or even half-metallic by substitutional nitrogen (N) and boron (B) atoms doping at the opposite edges. Recently, electrical transport measurements^29,30^ assented some inverse relationships between the band gap opening and the GNR width without corroborating evidence on the consequence of edge orientation. In an important breakthrough, Kim *et al.* demonstrated very large magnetoresistance values for graphene nanoribbons.^31^ The possibility of perfect spin-filtering device fabrication based on edge-functionalized graphene nanoribbons was also demonstrated recently.^32^

The recent scientific research conceivably showcase substantial developments to understand the existence of localized electronic states at zigzag edges of graphene.^33^ However, the theoretical predictions on the edge spin ferromagnetism are yet to be affirmed by some meticulous experimental assessments. It is important to note that the magnetic order on the zigzag graphene edges is evident from the transport and scanning tunneling microscopic measurements.^34,35^ Furthermore, these measurement schemes were based on inherent electronic charges (charge-based) and not on the electronic spin; in principle, they could be subjected to different approximations. In our previous study,^36^ we have probed the sequential changes in electronic and magnetic properties of ZGNR due to the simultaneous increase in the doping concentration (boron and nitrogen) and ZGNR width. In this particular study, we will paraphrase about the regulation of band gap and other electronic and magnetic properties of ZGNR by embedding an array of alternate boron and nitrogen impurities. Subsequently, we replaced a complete row of carbon atoms in the hexagonal network at different locations with an alternate boron and nitrogen sequence of doping. Deliberately, we averted doping at the zigzag edges of the system as it was extensively investigated in the last couple of years. It is important to note that we observed that it is possible to tune the band-gap as well as other electronic properties of the ZGNR according to the specific position of the substituted B–N arrays. Depending on the location of the doped B–N impurities, the ZGNR system showed the characteristics of either an insulator, a semiconductor or a semi-metal. This study will enlighten some important aspects of doping-induced band-gap engineering in zigzag GNRs. We presumed that our theoretical interpretations will account for a relatively simpler technique to control the electronic modulations in ZGNR and provide some useful insights to the application prospects of ZGNR as an exciting building-block (nanomaterial) for device fabrications.

## 2. Computational details

**Fig. 1:**
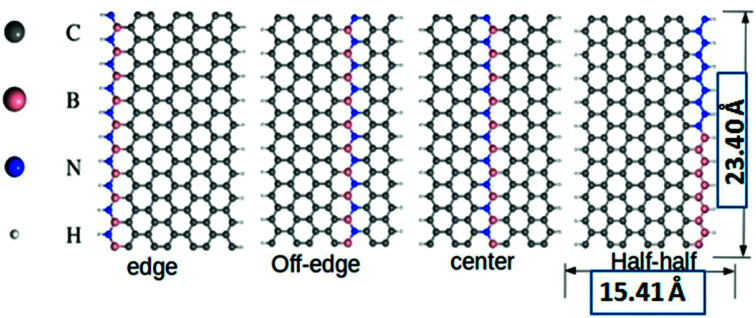
The typical pictorial representation of the model doped 7-ZGNR nanoribbon systems. All structures are relaxed at PBE level using Troullier and Martins norm-conserving pseudopotentials. Corresponding length and breadth of the nanoribbons are given accordingly.

All the DFT based *ab initio* calculations were performed using the open source Quantum Espresso 5.4.0 code.^37^ We used Troullier and Martins^38^ norm-conserving pseudopotentials and exchange-correlation (XC) effects were incorporated at the Perdew–Burke–Ernzerhof (PBE)^39^ level. The van der Waals correction to the calculations was incorporated through semi-empirical Grimme's DFT-D2 methodology^40^ as implemented in Quantum Espresso. Subsequently, we also tested the performance sensitivity of PBE level without incorporating the DFT-D2 correction. This test did not account for any significant changes to the computed parameters with the reproducible outcomes. The standard kinetic energy cutoff for wavefunctions and the corresponding cutoff for charge density and potential were set at 40 Ry and 160 Ry, respectively. We considered 4, 5, and 7-ZGNRs as the model systems for our study, where the particular numeral denotes the number of dimer lines across the zigzag edges. The structures of the model 7-ZGNR systems along with the corresponding doping patterns are included in Fig. 1 and pictorial representations of 4 and 5-ZGNR systems are included in ESI (S1).† The integration over the Brillouin zone was set as an optimized Monkhorst Pack^41^ of 1 × 1 × 10 *k*-points (GNR growth along the *z*-direction). The forces were minimized to <10^−3^ Ry a.u.^−1^ for all atoms to obtain fully relaxed atomic geometries. In the model systems, the dangling bonds at the edge atoms were saturated with atomic hydrogens. We adopted one-dimensional periodic supercells with 10 Å vacuum regions in both *x* and *y* directions to minimize the self-interaction between the repetitive units.

## 3. Results and discussions

Graphene-based materials open-up many new avenues due to their tunable electronic and magnetic properties. Inspired by this fact, our present study will focus on some deeper insights into the calculated parameters and their relevant scientific interpretations for the BN-doped ZGNR systems. It is worth mentioning here that the incorporation of B and N impurities in the hexagonal carbon network of ZGNR impart substantial changes to the inherent electronic environment.

Our current model systems comprise of four different types of BN-doping sequences, namely, edge, off-edge, center, and half–half. The corresponding modeled structures are reported in Fig. 1. In the edge doped system, a single row of hydrogen-terminated carbon atoms was replaced with an array of alternate B and N atoms in the ZGNR framework. Similarly, another row of carbon atoms away from the H-terminated edges was substituted with an alternate BN-atom array in the off-edge system. Again, we introduced BN impurities array in place of the carbon atoms at the center of the ZGNR in the central system. Subsequently, in the fourth system, the sequence of alternate BN-doping at one of the hydrogen-terminated ZGNR edges was replaced with an equal number of B and N substitutions in a continuous sequence. It is well established that the preferential doping sites for the ZGNRs are at the edges.^42,43^ However, our DFT based calculations predicted that the B and N doping not only at the zigzag edges but also at certain different locations induced effective modulations to the existing tunable electronic and magnetic properties of the ZGNR. A graphical representation of the relative energies of the four 7-ZGNR systems is given in Fig. S2 (ESI† section). The system containing doped B and N atoms at one of the H-terminated edges was found to be the most stable. Intuitively, considering the energy of the most stable system as a reference point, we plotted the relative energies of the other three doped ZGNRs accordingly. We observed that the half–half system with an equal number of B and N atoms in a continuous pattern at the H-terminated edges was significantly less stable as compared to the other three systems. Both off-edge and center systems showed comparable energies. Consequently, we can argue that the continuous electron–electron (NN) or hole–hole (BB) doping at the H-terminated edge of ZGNR was not energetically favorable. Hence, in our proceeding discussions, we will put limited emphasis on the half–half system. It is well known that the spin-dependent properties along with electronic transport properties of ZGNRs have a strong dependency on the width of the system. Although 7-ZGNR was our targeted system, to contemplate the relative changes in the electronic environment of ZGNRs instituted from the variable width, we also included the 4 and 5-ZGNR systems in our study. The structures and the doping sequences of these two systems are included in the ESI (S1).†

**Fig. 2:**
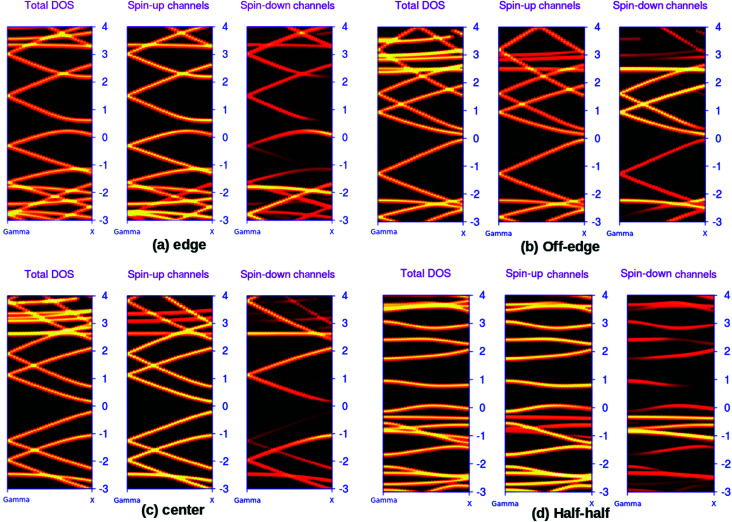
DFT based, calculated spin polarized electronic band structures for the four different BN-doped 7-ZGNR systems. Here, the three different panels for a particular system individually represent the overall, spin-up and spin-down electron distributions, respectively. The relative Fermi level is set at zero. The brightness of a particular band indicates the relative accumulation of electron densities. Here, brighter the color, the higher the electron densities and *vice versa*.

The possibilities of regulating the band structure of the BN-doped ZGNRs for the applications such as spin-dependent transport are well documented in the literature.^44,45^ Perhaps, the edge-decorated systems are the most extensively studied analogs of ZGNR. We reported the spin-resolved band structures of the four 7-ZGNR systems in Fig. 2. A considerable impact on the electronic environment due to the relative changes in the position of the doping impurities in ZGNRs carbon network is evident from the band diagrams. Indeed, the unusual electronic behaviors such as the half-metallic or semi-metallic characteristics of B–N doped ZGNR could be important aspects of the material in terms of its application in spintronics.^5^ However, our present study does not account for such peculiar electronic behaviors of the doped ZGNR systems. The spin degeneracy of the systems continues to exist and the up and down spin channels were coexisting in the overall band structure. This might be due to the sequential ordering of alternate electron–hole (B–N) doping in the structures. As we have emphasized in our earlier study, a lower level of impurity concentration (boron and nitrogen) at the ZGNR edge was primarily important to induce the half-metallic nature of the doped ZGNR system.^36^ It was observed that the introduction of an extended alternative B–N array at one end of the H-terminated edge of the ZGNR imparted some metallic characteristics to the system, which can be understood from the corresponding band structure (Fig. 2(a)). The significant increase in the density of states (DOS) around the Fermi level for hybrid BN-doped systems were well documented in some recent studies. It is important to note that our modeled systems were qualitatively similar to the hybrid nanoribbons structures reported by Yu *et al.*.^46^ The characteristic flat band at the Fermi level for the off-edge and center systems along with slight band-gap opening for the systems are consistent with their reported study. We have seen that the valence band top (VBT) for both spins up and down electron channels appeared to cross the Fermi level. In another situation, shifting the impurity substitution away from the H-terminated edges induced some distinct changes to the band structure of the system. Although the metallic characteristics in the ZGNR system are evident from Fig. 2(b), the conduction band minima are relatively close to the Fermi level as that of the above-mentioned situation. Here, the valence band top (VBT) and the conduction band minimum (CBM) meet at the Fermi level. This, to some extent, resembles a gapless semi-conducting state. Subsequent alternate BN array substitution at the center of the ZGNR carbon network led to an opening of the band gap in the system, as shown in Fig. 2(c). Similarly, when one of the H-terminated edges of the system was decorated with an equal number of B and N atoms in a continuous sequence, we observed a completely different band structure for the system. This introduced a distinct increase in the band gap of the system (Fig. 2(d)) with the appearance of flat bands near the Fermi level. The valence band top (VBT) and conduction band minimum (CBM) were aligned parallel to the to the Fermi level and positioned relatively far apart from one another, opening up a large energy gap as compared to that of the other three systems.

The above-mentioned systematic band structure analysis reveals some important aspects of the modeled BN doped ZGNR system. We have seen that it is possible to simulate different electronic environments in ZGNR by varying the position of the impurities in the hexagonal network. The band gap of the material can be regulated within a large energy window by tuning the position of the doping impurities array. In an important study, Xiao *et al.* also reported the possibilities of tuning the band-gap of ZGNR by alternately sequencing the BN-impurity array in the carbon network.^47^ However, the observed reduction in spin-degeneracy for some of the systems at the Fermi level according to their report was not consistent with our findings. The best possible explanation for this particular contradiction is the different arrangement of impurity centers in our study as compared to that of Xiao *et al.* We worked with a system containing a complete row of alternate boron and nitrogen atoms, while they incorporated doping atoms only to half of the carbon array and kept the remaining half unchanged. It is more likely that the incorporation of a single impurity in a specific position of hexagonal carbon network needs extreme precision along with high degree of sophistication. Presumably, the introduction of doping impurities into a complete row of carbon atoms is less complicated and more feasible.

**Fig. 3:**
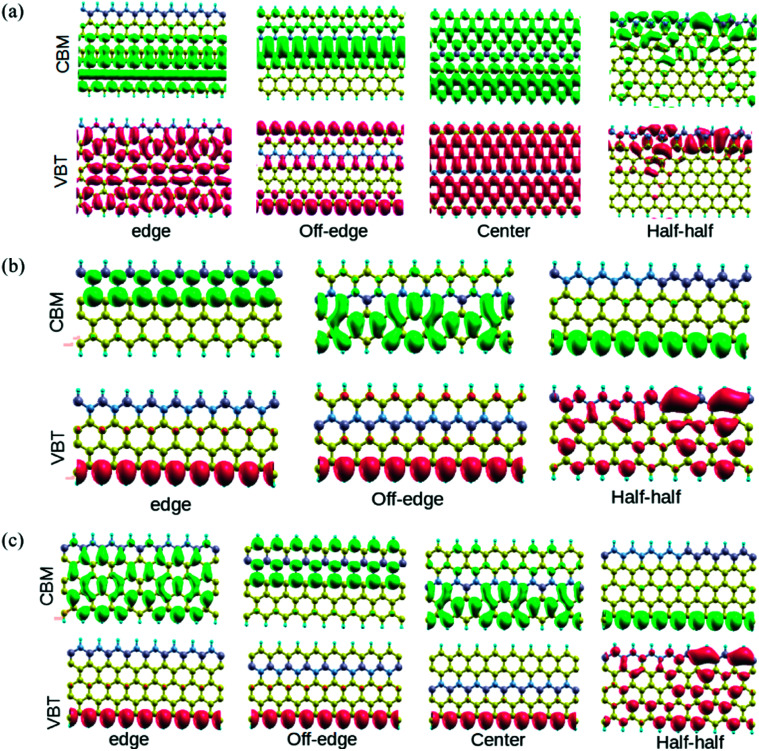
The highest occupied level (valence band top (VBT)) and corresponding lowest unoccupied level (conduction band minimum (CBM)) colored in red (lower panel) and green (above panel), respectively for the three BN doped ZGNR systems. (a) 7-ZGNR, (b) 4-ZGNR and (c) 5-ZGNR.

In order to understand the effect of alternate BN doping at different positions on the electronic structure of ZGNRs, we plotted the isosurface charge densities for VBT and CBM at their respective *k*-points (Fig. 3). It could be observed that the alternate BN substitutions at the edges of ZGNR led to some symmetrically distributed charge densities at the VBT in 7-ZGNR system. Except for the doped N atoms, all other atoms produced sound contributions to the VBT. However, there were no contributions from the doped impurities at the edge toward the CBM. In the off-edge doping, the VBT part was primarily located on the edge carbon atoms, indicating more or less a genuine ZGNR behavior, while the CBM was mainly localized on the central carbon atoms of the ZGNR network. Moreover, an array of alternate BN centers in the middle of the ZGNR network induced significant changes to the corresponding VBT and CBM wavefunctions. Similarly, the computed VBT and CBM wavefunctions for the 4 and 5-ZGNR systems are depicted in Fig. 3. In the 4-ZGNR system, the highest occupied states for edge and off-edge doped systems are located at the opposite edge to that of the doped system (Fig. 3(b)), while for the half–half system, it was mainly concentrated on one side of the system. Again, the CBM wavefunctions for all the three systems were different. A similar organized pattern for the highest occupied states is prevalent in 5-ZGNR systems. For the edge, off-edge and center systems it is concentrated at the carbon atoms of the hydrogen-terminated edges (Fig. 3(c)), while for the half–half system it is similar to that of the 4-ZGNR system. The lowest unoccupied states for the first three systems are spread over the entire system in various symmetric patterns. However, we did not observe any contributions from the doped impurity centers towards the FMOs in the case of both 4 and 5-ZGNRs, which is fairly consistent with the observations for the 7-ZGNR systems. It is worth mentioning that the width of the ZGNR systems has some minor impact on the highest occupied and lowest unoccupied states of the systems. It is likely the position and sequence of the doping impurities, which determined the degree of modulation induced to the electrons present in FMOs of a particular ZGNR system.

**Fig. 4:**
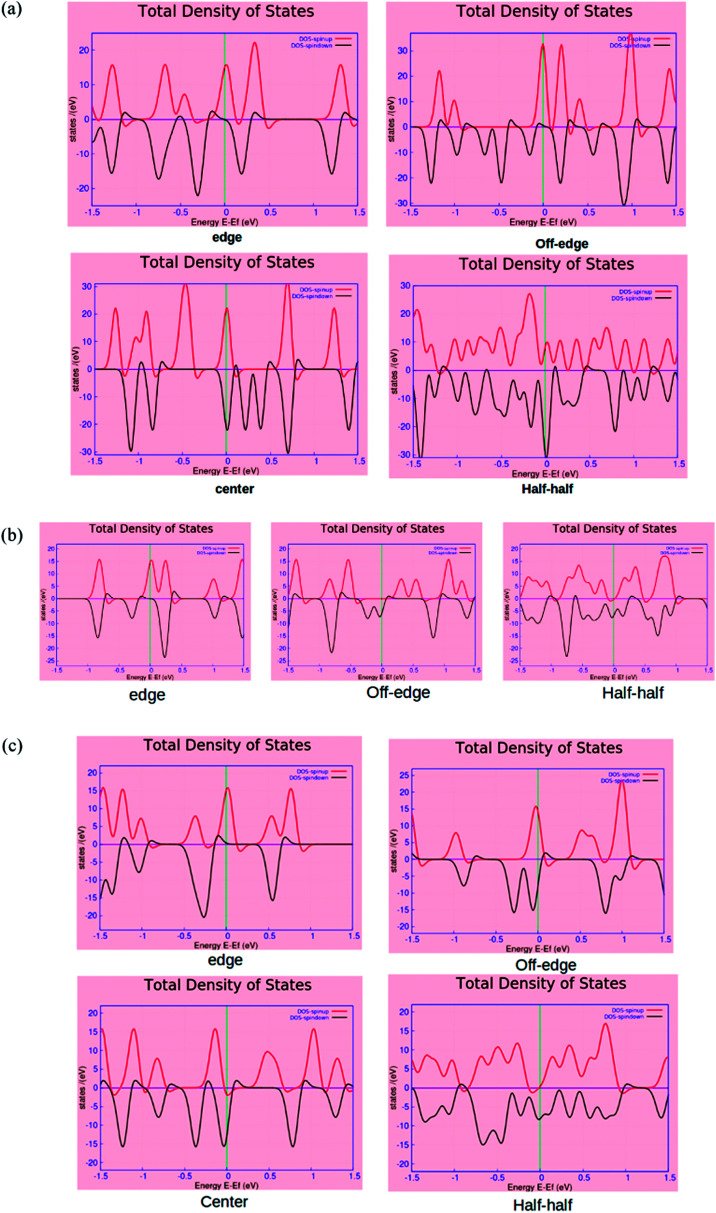
The spin polarized total density of states (TDOS) plots for the (a) 7-ZGNR, (b) 4-ZGNR and (c) 5-ZGNR systems. The red and green lines represent the spin up and down electron configurations, respectively. The Fermi level sets at zero.

The spin-polarized electronic density of states (DOS) plots for the various BN-doped ZGNR systems are reported in Fig. 4, where (a), (b) and (c) are the DOS plots for 7, 4, and 5-ZGNR, respectively. The distinct changes in the properties of up and down spin electrons according to the location of BN impurity array can be understood from the DOS spectrum. The calculated DOS spectrum exhibited relatively strong peaks (either up or down spin electrons) positioned at the Fermi level of all four systems. In the case of ZGNRs, these sharp peaks primarily accounted for the contributions of pi-electrons in the edge states. This particular electronic property in ZGNR systems is claimed to be the consequence of quantum confinement effects and is reportedly sensitive to the width of ZGNR.^48^ We observed that the position of electronic states belonging to the edges in the DOS spectrum could be regulated by tuning the impurity locations in the carbon network. It is worth mentioning that relative to the Fermi level, the two spin states appeared to have some distinct shifts in the opposite directions and it was consistent with the previous reports.^22^ Similar results emphasizing the appearance of a flat band near the Fermi level encountered due to the presence of edge state were also evident from some earlier studies.^49,50^ More precisely, flat band is the consequence of localized state at the edges, which leads to the occurrence of intense peaks near the Fermi level in the local density of states (LDOS) for the edge carbon atoms. It could be argued that due to the incorporation of doped impurities, the edge states encounter some distinct shifts around the Fermi level, which is contrary to that of the pristine ZGNRs. We have observed almost similar electronic energy states for the 7 and 5-ZGNR systems as represented in the DOS spectrum. The metallic nature along with strong spin polarities in these systems were also accounted from their respective DOS spectrum. It can be argued that the variation in the width of the ZGNR does not have a significant impact on the electronic structure, specifically for the edge doped systems. This can be realized from the computed DOS structure for the edge doped systems. The off-edge doping brought about some distinct changes to the DOS spectra of the three different modeled ZGNR systems. We observed that the peaks positioned at the Fermi level of 5-ZGNR shifted to the lower energy side relative to that of the 7-ZGNR. This also indicated some reduction in the spin polarity of the 5-ZGNR. However, the 4-ZGNR system exhibited a completely different order of electronic states energies for the off-edge doping situation. It is well understood from the above discussions that the width as well as the location of doping impurities in the hexagonal carbon network has a significant impact on the electronic structures of graphene nanoribbons.

The visual representation of the computed isosurface plots of the local density of states (LDOS) for the different ZGNR systems is available in the ESI (Fig. S3†). It is important to note that the variation in the GNRs width also introduced changes to the LDOS distribution patterns in the system. It is clear from the plots that an alternate sequence of B–N impurities at one of the hydrogen-terminated ZGNR edges modulated the usual local density of states of the system to a significant extent. In that situation, the LDOS was mostly concentrated on the doped edge for the 7-ZGNR. In the 4-ZGNR system, the edge doping produced almost equal LDOS distributions at the two H-terminated edges. However, for 5-ZGNR with alternate BN-doping at one of the H-terminated edges, the LDOS seemed to be spread across the system. Indeed, the presence of BN-impurities away from the hydrogen-terminated edges accounted for some substantial changes in the symmetric distribution of LDOS in the ZGNR system as depicted in the Fig. S2.†

**Fig. 5:**
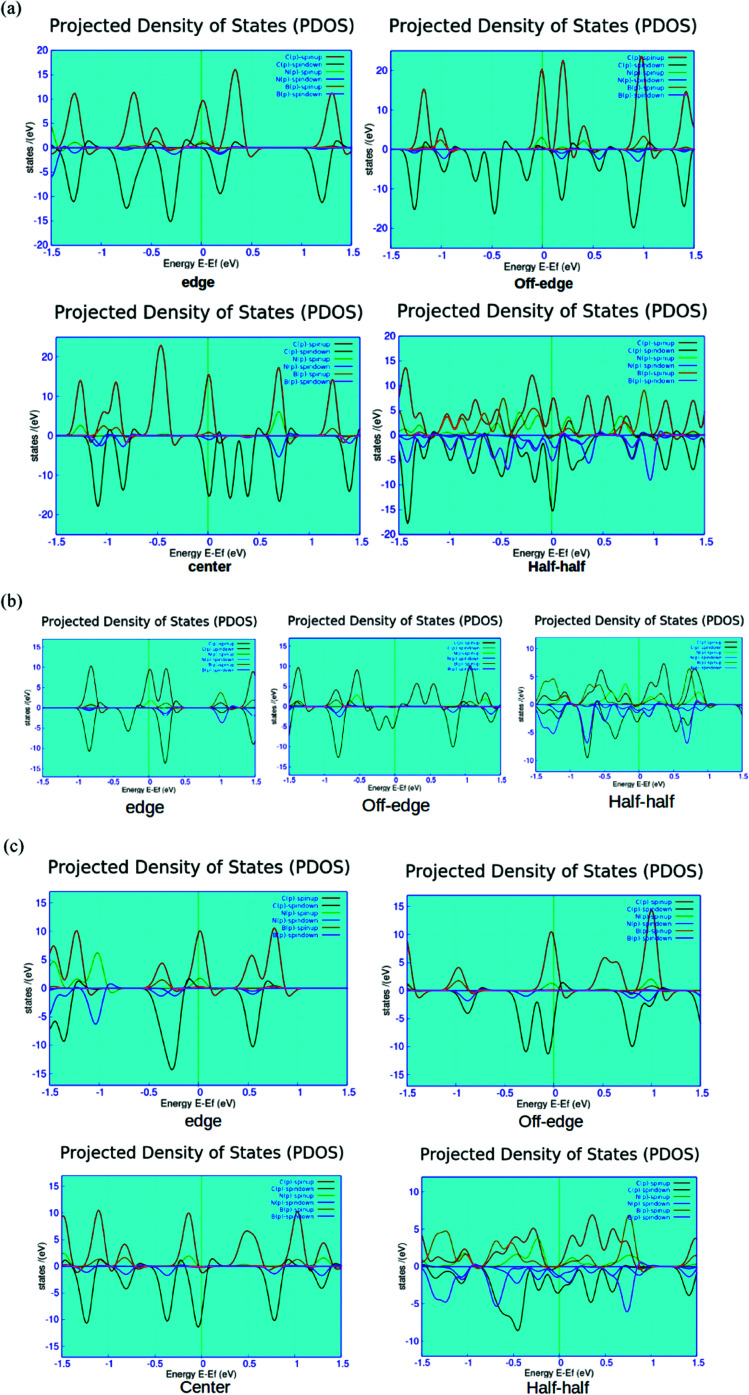
Computed spin polarized partial density of states (PDOS) plots for the four-different doped (a) 7-ZGNR, (b) 4-ZGNR and (c) 5-ZGNR systems. Here, the red (spin-up) and green (spin-down) lines represent the p-electrons' contribution from the edge carbon atoms. The light blue (spin-up) and maroon (spin-down) are the electrons' contribution from the doped nitrogen atoms present in the system. The yellow and blue lines exhibit the contributions of spin up and down electrons, respectively from the doped boron impurities.

We report the computed spin-polarized partial density of states (PDOS) plots for the four different BN-doped ZGNR systems in Fig. 5. As shown in the figures, the red and green lines depict the density of spin up and down p-electrons of carbon atoms at the zigzag edges. This effectively indicated the solo contribution of the edge state carbon p-electrons to the overall density of states (DOS) of the system at the Fermi level. Such findings well-agree with the earlier reports.^12,51,52^

As shown in Fig. 5, the system containing alternate BN-doping at the H-terminated edges exhibits minor contributions from the doped B and N impurities below the Fermi level. The edge state doping is consistent with all the three different variants of ZGNRs (*i.e.*, 4, 5 and 7-ZGNRs). It can be argued that the width of ZGNR does not impart major changes to the electronic environment of the system accounted from the impurity doping at the hydrogen-terminated edge. However, the off-edge doped system showed the partial development of impurity states above the Fermi level for 7 and 5-ZGNR systems. Interestingly, in the case of 4-ZGNR, we observed an entirely different situation. In this case, there is a significant improvement in the contributions from the down-spin electrons of edge carbon atoms at the Fermi level. As shown in the PDOS figure, the introduction of BN-impurities at the center of the ribbon does not have much impact on the overall DOS spectrum except for some minor growth in the contributions from the doped states far below the Fermi level for the 7-ZGNR. Again, for the 5-ZGNR system, we observed some distinct shifts in the edge carbon atom peaks located at the Fermi level toward the lower energy. The observed characteristic up-spin nitrogen impurity peak near the Fermi level for 5-ZGNR did not appear for the 7-ZGNR; instead, there was a minor growth in the contributions from both doped boron and nitrogen atoms at the Fermi level. Similarly, the half–half system exhibited some unusual features. The relative atom contributions toward the overall DOS for 4 and 5-ZGNRs were almost similar. At the Fermi level, associated DOS was the combined contributions of down-spin p-electrons from carbon, boron and nitrogen atoms. Moreover, in 7-ZGNR at the Fermi level, p-electrons of edge carbon atoms were the main contributors to the spin-up channels. We have also realized relatively symmetric electron density patterns, followed by the substantial reduction in spin-polarity of the system. However, the p-electrons of nitrogen showed a significantly higher contribution at the spin-down channels, followed by the p-electrons of carbon and boron atoms, respectively.

It is well known that N doping introduces an extra electron to the ZGNR network, while B doping provides a hole. As we have seen from the PDOS plots, the position of the impurity state was varied according to the location of BN array in the network. The extra electron prefers to stay in the vicinity of N^+^ state. Basically, the position of impurity states in the energy level depends on two important factors. First, the existing equilibrium between the coulombic interaction between the N^+^ and unpaired electron and the dynamic correlation of unpaired edge state p-electrons with the extra non-bonding electron in N atom. Second, the relative distance between the nitrogen atom and the ribbon edge. The presence of nitrogen impurities at one of the H-terminated edges led to some strong interactions between the extra non-bonding electron and N^+^ states. Consequently, the impurity states appeared below the Fermi level. However, when the nitrogen impurities were located away from the edges, the electronic correlation between the non-bonding electron and the edge-state p-electrons became the dominating factor and the non-bonding electron was preferably located at the edge atoms. It is noteworthy that N^+^ has a strong tendency to attract pi-electrons from the surrounding carbon atoms, thus forming a negative electron cloud around the central N^+^. This particular phenomenon is responsible for the occurrence of impurity states above the Fermi level in the off-edge system.

**Fig. 6:**
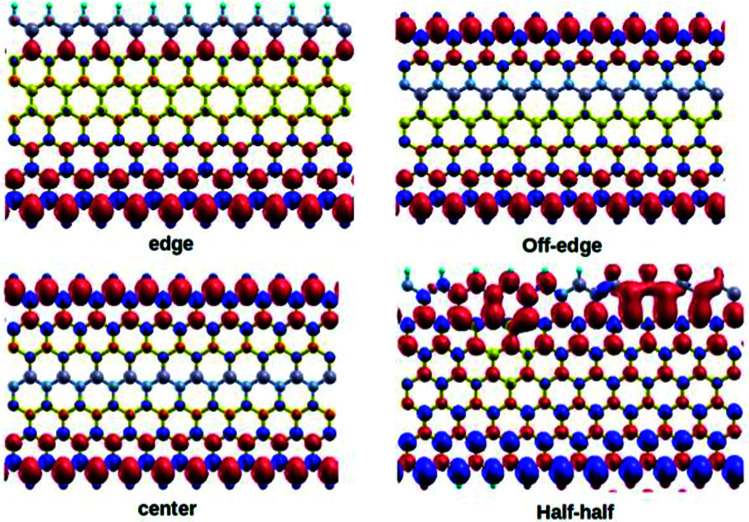
The spin density distribution plots for the four different BN-doped ZGNRs. The red and purple color regions in the plots represent the oppositely aligned excess spin densities.

Currently, the spin-dependent magnetic properties of the zigzag graphene nanoribbon are one of the widely investigated topics in material research. The tunable magnetic properties of doped ZGNRs generated a great enthusiasm among the researchers. Although a detailed discussion on magnetism of this particular form of carbon material is beyond the scope of the present paper, we will focus on some of the important aspects of magnetism observed in our modeled systems. Subsequently, our DFT calculations predicted that the magnetic moments for the different 7-ZGNR systems were in the range 1.00–1.60 bohr magneton per cell. Interestingly, we observed an increase in the total magnetic moment values with the decrease in the width of ZGNR systems (*i.e.*, 4 and 5-ZGNRs). The computed values were in the ranges of 0.00–2.00 and 1.90–2.30 bohr magneton per cell for 4 and 5-ZGNR systems, respectively. Fig. 6 represents the computed isosurface spin density distribution plots for the 7-ZGNR systems. Similar spin density representations for the 4 and 5-ZGNR systems are included in the ESI section (S4†).

It is worth mentioning that the position of doped BN-arrays do not substantially modulate the magnetic behaviors of the systems. There were some minor variations to the overall magnetic moments of the systems. The anti-ferromagnetic ordering of the spin alignments in the two opposite edges was consistent with those in the off-edge and center systems. Consequently, the BN impurity doping at these two particular positions of the network would not strongly perturb the overall electronic spin alignments of the system. It might be possible that the local magnetic perturbations, induced due to the presence of alternate boron and nitrogen centers, are compensated by the magnetic field generated from the surrounding carbon atoms in the system. Hence, the usual spin polarization in the ZGNR systems continued to persist at the hydrogen-terminated edges. However, when the impurity substitution was introduced at the edges, the magnetic behavior of the system was disturbed to a significant extent. As observed for the 7-ZGNR, the introduction of an alternate BN impurities array at one of the H-terminated edges destroyed the characteristic anti-ferromagnetic spin orientations along the edges and led to the development of some strong ferromagnetic interactions on the carbon atoms next to the edges. However, the 4 and 5-ZGNR systems created some different impressions for the magnetic orientation in the edge doping. As we have seen, the calculations for 4-ZGNRsuggested the evolution of strong ferromagnetic interactions between the doped centers along the edge. Moreover, 5-ZGNR exhibited a significant reduction in the spin magnetic states due to BN-doping along the edge. At this point, it can be argued that the particular orientation of the doping sequence at a certain location of the hexagonal carbon network can efficiently regulate the spin-dependent magnetic properties of the system. We have also noticed that the width of ZGNR strongly influenced the magnetic behavior of the system. In principle, the sequence of impurity doping and width of the system were the exclusive measures to engineer the electronic and magnetic properties of ZGNR for nanoelectronics applications.

## 4. Conclusions

In summary, using a DFT based approach, we assayed the possibilities of tuning electronic and magnetic properties of ZGNRs by substituting a complete row of carbon atoms with an alternate sequence of B and N impurities. The important point is that we can induce and regulate the electronic environment of the zigzag GNR not only by incorporating chemical modifications at the zigzag edges, but also by implementing some intuitive modeling on the hexagonal carbon network. Our calculations demonstrated that the site-specific array substitutions (BN array) rendered a comprehensive way to engineer the strong edge-orientated properties of the ZGNR systems. Furthermore, it may be possible to obtain an unprecedented control over both electronic and magnetic behaviors of the ZGNR systems through chemical modifications at the hydrogen-terminated edges, which was a novel aspect of our study. Eventually, the relative shift in the position of alternate BN-doping sequence regulates the band-gap of the system either from metallic to semiconducting or insulating states. In particular, these array substitutions did not break the spin-degeneracy of the system, so we had the freedom to tune the electronic behavior without affecting the spin-dependent properties. Indeed, a systematic control of the rich electronic and magnetic properties of the zigzag graphene nanoribbon in terms of sustained chemical modifications will find some potential applications in the nanoscale electronic devices.

## Conflicts of interest

There are no conflicts of interest to declare.

## Supplementary Material

RA-008-C8RA00386F-s001
