# Supplementary material for: Sequential BN-doping induced tuning of electronic properties in zigzag-edged graphene nanoribbons: a computational approach
Source: RSC Adv. 2018 Mar 19;8(20):10964–74. doi: 10.1039/c8ra00386f (PMC9078980; doi:10.1039/c8ra00386f)
Supplement: RA-008-C8RA00386F-s001 [file RA-008-C8RA00386F-s001.pdf]

Electronic Supplementary Material (ESI) for RSC Advances.
This journal is © The Royal Society of Chemistry 2018

Electronic Supporting Information

Sequential BN-doping Induced Tuning of Electronic Properties in Zigzag-edged Graphene Nanoribbons: A Computational Approach

Amrit Sarmah,[a,*] Pavel Hobza[a,b]

[a]Institute of Organic Chemistry and Biochemistry of the Czech Academy of Sciences, Flemingovo nam. 2, CZ-16610 Prague 6, Czech Republic

[b]Department of Physical Chemistry, Palacky University, CZ–77146 Olomouc, Czech Republic

Table of contents

S1 Pictorial representations of the BN-doped 4 and 5-ZGNR systems.
S2 Graphical representations of the relative energies for the relaxed structures of doped graphene nanoribbons.
S3 The visual representation of the computed local density of states (LDOS) for the different ZGNR systems
S4 Spin density isosurface plots for the 4 and 5-ZGNR systems considered for our present study.

*Corresponding Author
E-mail Address: amrit.sarmah@marge.uochb.cas.cz (Amrit Sarmah)

(a)

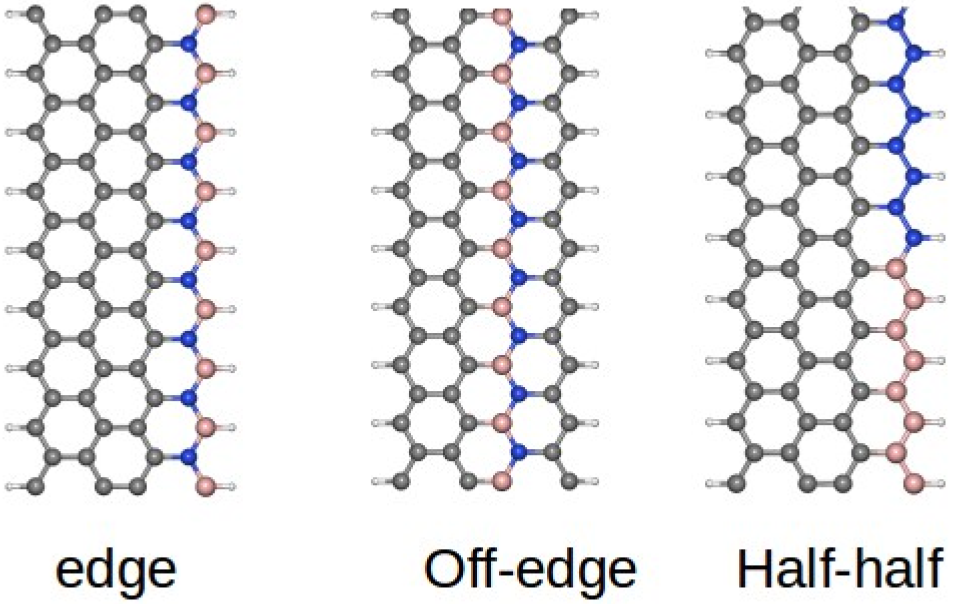

(b)

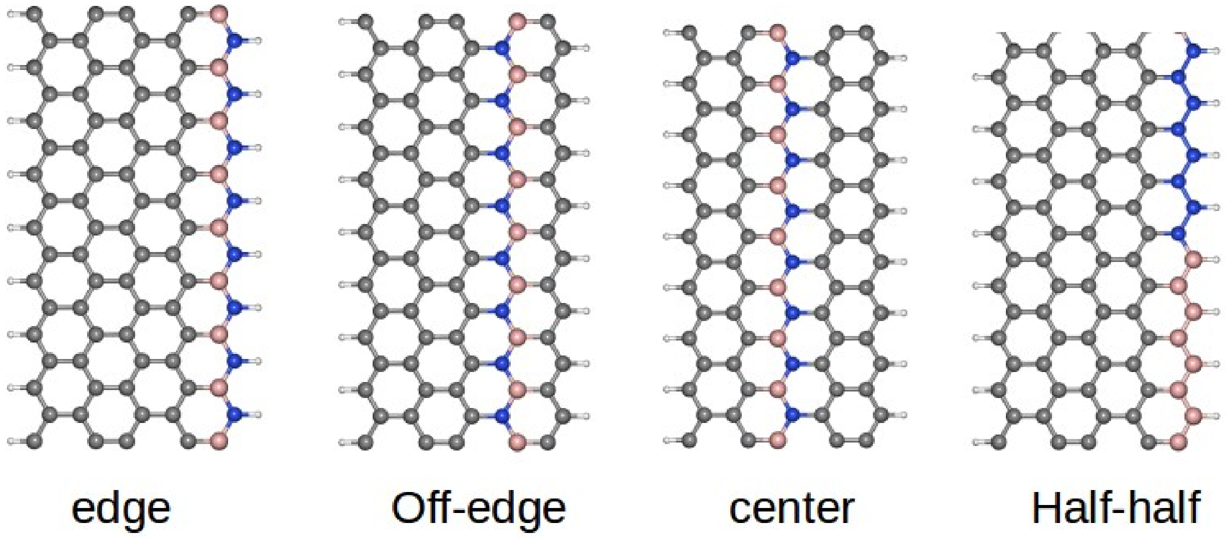

Fig. S1. The typical pictorial representation of the model 4 (a) and 5-ZGNR (b) systems and their corresponding doping pattern.

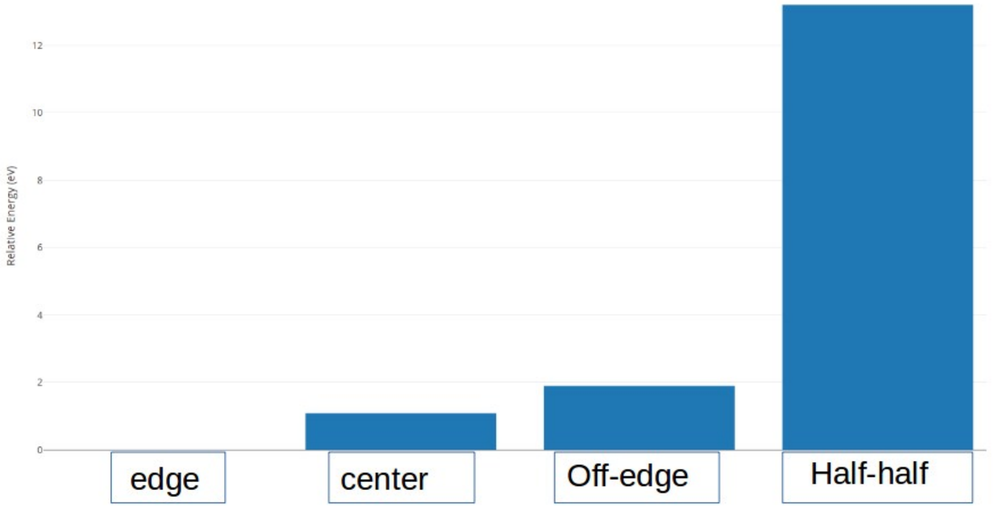

Fig. S2. A graphical representation of the relative energy values for four BN doped nanoribbon systems used in our present study. Here, the most stable system i.e., edge substituted ZGNR system is taken as the reference point and the other energies are plotted with respect to the reference system. Higher energy corresponds to lower stability.

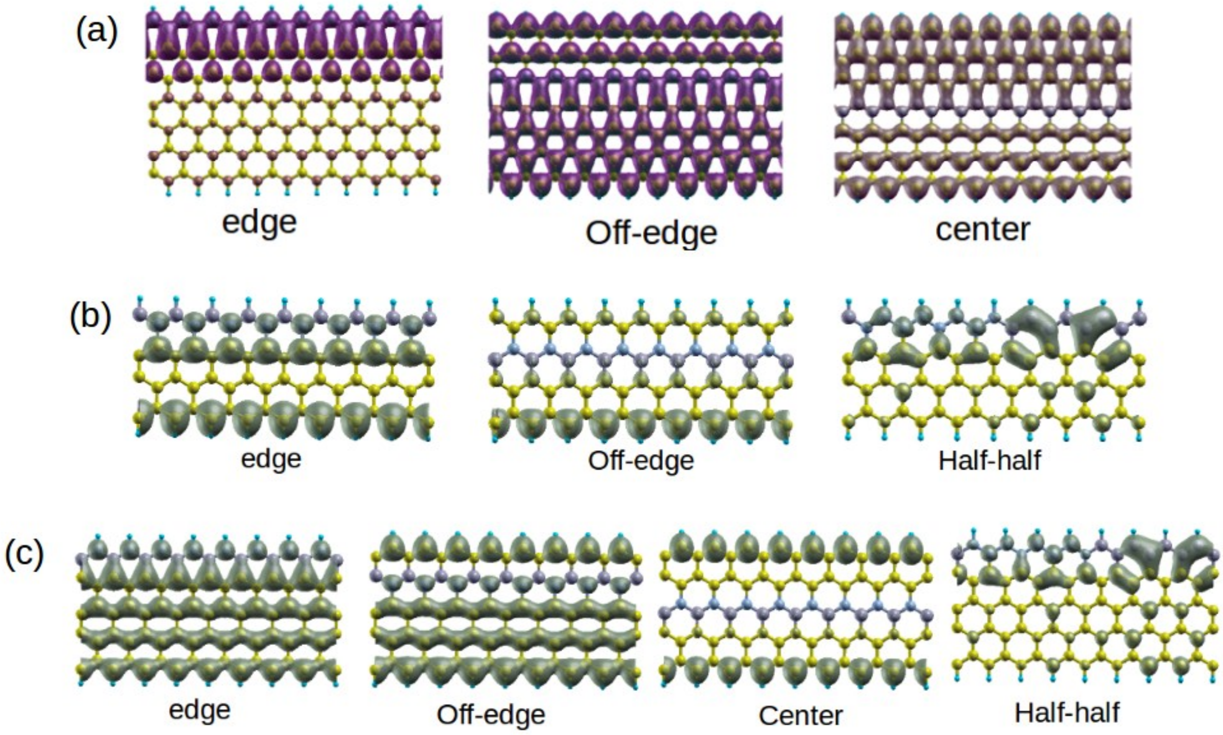

Fig. S3. The visual representation of the computed isosurface map for the distribution of local density of states (LDOS) for three BN-doped ZGNRs. (a) 4-ZGNR, (b) 5-ZGNR and (c) 7-ZGNR systems.

(a)

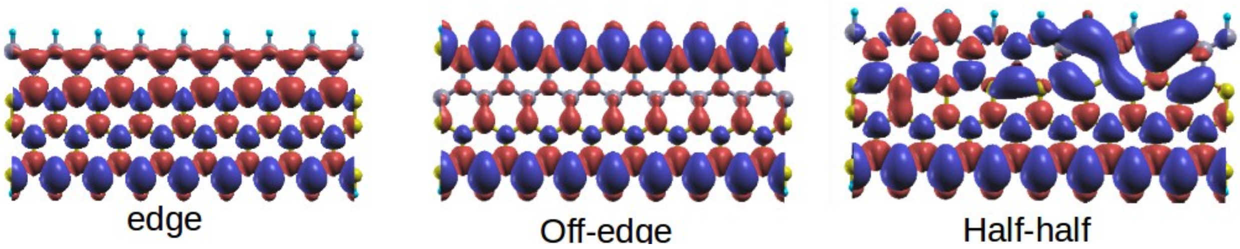

(b)

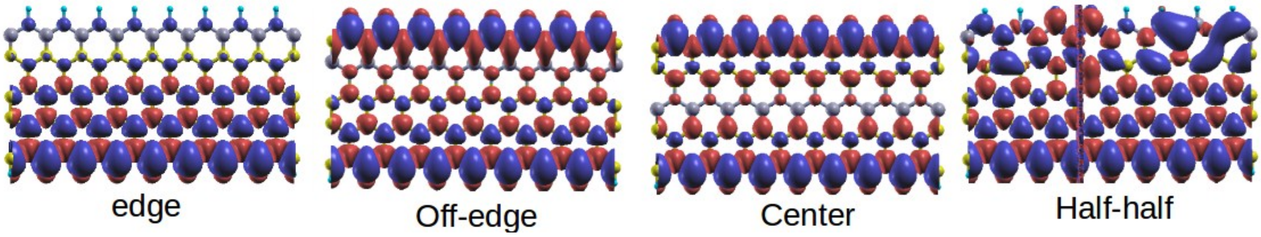

Fig. S4. The spin density distribution plots for the different BN-doped 4 (a) and 5-ZGNRs (b). The red and purple color regions in the plot represent the oppositely aligned excess spin densities.
